# Magnetic resonance imaging in canine idiopathic epilepsy: a mini-review

**DOI:** 10.3389/fvets.2024.1427403

**Published:** 2024-07-03

**Authors:** Kari D. Foss, Audrey C. Billhymer

**Affiliations:** Department of Veterinary Clinical Medicine, College of Veterinary Medicine, University of Illinois Urbana-Champaign, Urbana, IL, United States

**Keywords:** MRI, epilepsy, dog, neuroimaging, brain, seizure

## Abstract

Magnetic resonance imaging (MRI) in an integral part of the diagnostic workup in canines with idiopathic epilepsy (IE). While highly sensitive and specific in identifying structural lesions, conventional MRI is unable to detect changes at the microscopic level. Utilizing more advanced neuroimaging techniques may provide further information on changes at the neuronal level in the brain of canines with IE, thus providing crucial information on the pathogenesis of canine epilepsy. Additionally, earlier detection of these changes may aid clinicians in the development of improved and targeted therapies. Advances in MRI techniques are being developed which can assess metabolic, cellular, architectural, and functional alterations; as well alterations in neuronal tissue mechanical properties, some of which are currently being applied in research on canine IE. This mini-review focuses on novel MRI techniques being utilized to better understand canine epilepsy, which include magnetic resonance spectroscopy, diffusion-weighted imaging, diffusion tensor imaging, perfusion-weighted imaging, voxel based morphometry, and functional MRI; as well as techniques applied in human medicine and their potential use in veterinary species.

## Introduction

Epilepsy is one of the most common chronic neurologic conditions in dogs with an estimated prevalence of 0.62–0.75% in the general dog population (1–3). The diagnosis of idiopathic epilepsy (IE) is based on the exclusion of other underlying etiologies for which magnetic resonance imaging (MRI) is essential to the diagnostic workup and considered part of the Tier II confidence level for the diagnosis of IE as put forth by the International Veterinary Epilepsy Task Force (4). Three main aims of advanced MRI in the epileptic animal are: (1) to rule out causes of epileptic seizures which may be treatable with means other than antiseizure medications (ASM) only (e.g., inflammatory or infectious brain disease), (2) to identify lesions caused by epileptic seizures which are not themselves the source of seizures, and (3) to provide data to further advance the field of research into the pathogenesis and/or treatment of epilepsy (5).

However, conventional, or structural MRI lacks the specificity to identify many disease processes due to significant overlap in imaging characteristics and lesion morphology between intracranial etiologies in dogs (6, 7). As such, the study of idiopathic (or “non-lesional” in human medicine) epilepsy with MRI is challenging as this condition often presents with a normal-appearing brain (8). Structural abnormalities are identified in only 2.2% of dogs less than 6 years of age with epileptic seizures (9). There are reports of visible, or invisible, but statistically identifiable findings in canine epilepsy. Visible post-seizure changes have been documented in both idiopathic and structural epilepsies and can include regions of T2W or fluid attenuated inversion recovery (FLAIR) hyperintensity and T1W iso-to hypointensity with variable contrast enhancement, with the majority of these changes being bilateral and symmetric (9–14). Structures specifically involved include the hippocampus, cingulate gyrus, and piriform lobe (10–12). These changes are often transient, and likely represent a combination of cytotoxic and vasogenic edema associated with increased energy metabolism, hyperperfusion, and cell swelling as a consequence of the ictal activity, but they do not represent the area of the cortex from which seizures arise and propagate, called the epileptogenic zone (8, 10–12).

The concept of the epileptogenic zone was initially proposed in people by Lüders et al. (15), and has since been proposed in canine epilepsy to be defined as “the region of cortex that can generate epileptic seizures and removal or disconnection of which should lead to seizure freedom,” and cannot be identified on routine anatomical imaging (8, 16). The epileptogenic zone consists of five different abnormal cortical zones: the symptomatogenic zone, the irritative zone, the seizure-onset zone, the structural abnormal zone (epileptogenic lesion), and the functional deficit zone (8). The functional deficit zone is defined as “the area of the cortex that is functionally abnormal in the interictal period.” This zone not only relates to structural (MRI visible) lesions but also to microstructural lesions and true areas of functional abnormalities, as occur in IE (8). Therefore, imaging modalities able to detect the epileptogenic zone are both essential for accurate presurgical evaluations and helpful in understanding the pathophysiology of canine and feline epilepsy.

In addition to identifying non-structural changes that may aid in the identification of the epileptogenic zone, there is growing evidence of structural changes within the hippocampus, temporal lobes, or white-to-gray matter ratios in dogs with IE which do not correlate with epileptogenic zone (17). As such, the International Veterinary Epilepsy Task Force published a consensus statement with recommended MRI protocols in the diagnosis of canine IE which may allow the detection of subtle lesions not apparent with existing sequences, imaging planes, or particular techniques. The developed protocol aims to facilitate improved evaluation of areas susceptible to the generation and perpectuation of seizures (5).

Advances in MRI techniques may also aid more detailed examination of these areas, including the ability to assess metabolic, cellular, architectural, and functional alterations, as well as alterations in tissue mechanical properties (18–20). This mini-review focuses on novel MRI techniques being utilized to better understand canine epilepsy, which include magnetic resonance spectroscopy (MRS), diffusion-weighted imaging (DWI), diffusion tensor imaging (DTI), perfusion-weighted imaging (PWI), voxel-based morphometry (VBM), and functional MRI (fMRI); as well as techniques applied in human medicine and their potential use in veterinary species.

## Techniques

### Diffusion weighted imaging and diffusion tensor imaging

**Figure 1:**
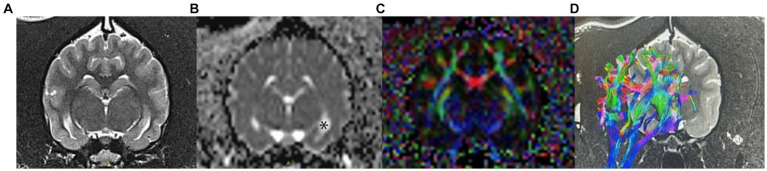
Examples of DWI and DTI images obtained in a healthy dog. **(A)** conventional T2-weighted image at the level of the hippocampus; **(B)** apparent diffusion coefficient (ADC) map; areas of unrestricted water diffusion, such as CSF in the lateral ventricles (*), have a high signal intensity on ADC maps. Additionally, gray matter has a higher ADC than white matter **(C)** fractional anisotropy (FA) color map measuring changes in water diffusion within the white matter tracts; **(D)** tensor tracking algorithm of the fibers of the corona radiata. Red indicates right–left direction, green indicates dorsoventral, and blue indicates a rostro-caudal direction.

Diffusion-based MRI, such as diffusion-weighted imaging (DWI) and diffusion tensor imaging (DTI), can identify variation in diffusion characteristics of intra-and extracellular water compartments, and exchange of water across permeable boundaries, based on the sensitization of the Brownian motion of water molecules. In the brain, the movement of water molecules in three compartments contributes to the measured degree of diffusion: intravascular perfusion, extracellular diffusion, and intracellular diffusion (21, 22). When this movement of water molecules is obstructed, it becomes anisotropic, or directionally dependent (23, 24). Assessing this anisotropy allows a significant amount of data indexes to be generated, including fractional anisotropy (FA) and apparent diffusion coefficient (ADC) (24, 25). Fractional anisotropy represents the amount of diffusional asymmetry in a voxel, and is a marker of white matter integrity (22, 26). Thus, it is a highly sensitive measure of the microstructural integrity of fibers. ADC represents the average magnitude of molecule displacement at any diffusion direction determined, and can be utilized as a marker of pathologic tissue changes (22) (Figures 1A–C).

Epileptic seizures have been shown to induce cytotoxic edema through excitotoxicity and thus, DWI is highly sensitive to neuronal damage secondary to seizure activity. Therefore, this technique is being applied in human and veterinary medicine to provide additional information regarding ongoing pathologic changes secondary to seizure activity (8, 22). A reduction in diffusion in human patients with temporal lobe epilepsy (TLE) has been described during the ictus and postictally, whereas increased diffusion has been observed in cases of suspected hippocampal sclerosis (21, 27, 28).

Studies in veterinary medicine have employed diffusion MRI techniques to aid in the identification of the epileptic zone or to identify areas of potential brain damage secondary to epileptic seizure activity (8, 21, 29). A study by Hartmann et al. assessed the feasibility of interictal DWI in dogs with IE to assess the distribution of diffusion in comparison to healthy dogs. Significantly increased diffusion was found in the piriform lobe (including amygdala) of epileptic dogs, proposed to be secondary to the loss of structural organization and expansion of extracellular spaces (21). These findings further support that there are changes at the cellular level in the brains of epileptic dogs. While DWI may be a promising technique in the evaluation of epileptic dogs lacking gross MRI abnormalities, these changes are likely transient. Experimental data in humans shows that ADC changes on MRI may last for a few days with the diffusion normalizing in most cases (27, 30, 31). Indeed, in an experiment of kainic acid-induced complex partial status epilepticus in dogs, a reduction in ADC was noted 3 and 6 h post-induction with increased diffusion after 12 and 24 h and normalization 48 h later (29). Therefore, the addition of imaging techniques that may detect more long-standing cellular changes is warranted as part of the diagnostic imaging assessment in dogs with epilepsy.

Similar to DWI, diffusion tensor imaging (DTI), enables the quantification of diffusion of water, but also allows the characterization of the degree and direction of anisotropy (32, 33). Following the collection of this data, tensor maps are generated and tractography can be performed. DTI is a method where connectivity maps between adjacent voxels are linked if the tensors are oriented in the same direction (23, 24, 34). Colors are then assigned to nerve fiber tracts depending on the direction of water displacement and this fiber tracking allows depiction of white matter tracts; comparison between normal and diseased fiber tracts enables quantification of white matter changes due to damage (35). Typically, the colors represent the predominant orientation of the fibers in a three-dimensional coordinate system along the axes of space (x, y, and z); red indicates right–left direction, green indicates dorsoventral, and blue indicates a rostro-caudal direction (Figure 1D). DTI has been applied in human medicine in the assessment of TLE, and has shown asymmetry in anisotropy as well as abnormalities not restricted to the temporal lobe (33, 36). In veterinary medicine, DTI has been utilized to establish the white matter tracts in the canine (and feline) brain (37, 38). However, there is only one study on the application of DTI in canine epilepsy. Beckmann et al. performed DTI in dogs affected by IE suffering from generalized tonic–clonic seizures to determine whether white matter diffusion is altered in these patients. The findings of this study showed subtle changes in DTI between dogs with IE and healthy dogs, particularly in the cingulate gyrus. In people with TLE, those suffering from generalized epilepsy syndrome have less pronounced changes than those with TLE. Therefore, these changes may also be less pronounced in dogs with generalized tonic–clonic seizures. The pathophysiology of these white matter changes is unknown, and may represent either a change occurring before the development of epileptic seizures, or a secondary effect (26).

### Magnetic resonance spectroscopy

**Figure 2:**
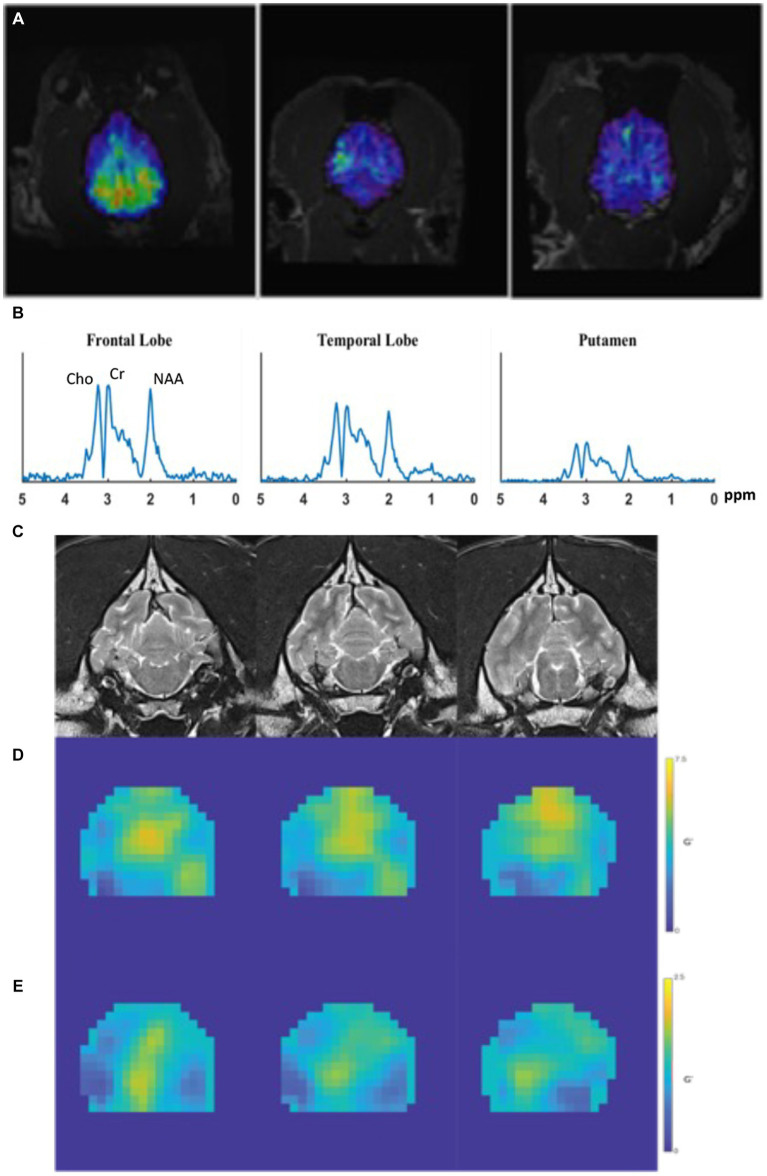
Examples of MRS and MRE performed in a healthy canine brain. **(A)** Dorsal MPRAGE of metabolite maps (NAA, overlaid on the corresponding anatomical MRI images) from a control dog demonstrating volumetric metabolite mapping. **(B)** Regional spectra from a dog showing peaks of Cho, Cr, and NAA from various brain regions. **(C)** T1-weighted coronal images obtained prior to MRE acquisition which are used for mapping of elastograms; **(D,E)** quantitative stiffness maps (elastograms) of the **(D)** shear modulus, and **(E)** loss modulus. In both **(D,E)**, areas of highest stiffness are yellow and areas of low stiffness are blue.

Magnetic resonance spectroscopy (MRS) is a noninvasive technique that provides quantitative measurement of specific metabolites in the brain, and may detect alterations before structural changes are observed (17, 39–41). These metabolites can be identified at 1.5 and 3 Tesla (T) MRI and include: include N-acetyl aspartate (NAA), choline (Cho), creatine (Cr), myoinositol (mI), and glutamate (Glu) and gamma-amino-butyrate (GABA) (40, 41) (Figures 2A,B). The dominant peak in the normal human brain spectra is NAA, which is restricted within neurons, axons, and myelin (42, 43). Therefore, it is considered a neuronal marker for brain neuronal health, viability, and number of neurons (17, 39, 42, 43). Choline (cho) is involved in the synthesis and decomposition of cell membranes and is therefore a marker for membrane damage and gliosis (23, 43). Creatine (Cr) plays a role in energy metabolism and while it is found in all types of neuronal cells, it is in greatest concentration within glial cells (43). Myoinositol (mI) is a glial marker and increased levels indicate glial changes and osmolarity disturbances (43). Glutamate and glutamine peaks overlap and are often measured together as glutamate-glutamine complex (Glx) (17, 44, 45). Increased levels of Glx have neurotoxic potential (23, 39). Metabolites that can be identified at 3 T and are of specific interest in epilepsy are glutamate, GABA, and NAA (39, 46).

In veterinary medicine, MRS has been used to determine the effects of ketamine on brain, specifically thalamic, metabolites in dogs with IE (44) and in the assessment of post-ictal metabolite changes in a generalized seizure model (47). Currently, there are two prospective studies which utilized MRS to evaluate metabolic changes in dogs with IE (17, 39). Olszewska et al. evaluated the interictal metabolic activity of the temporal lobe in dogs with IE compared to healthy controls at 1.5 T. NAA-to-choline (NAA/Cho), choline-to-creatine (Cho/Cr), and choline-to-NAA (Cho/NAA) ratios were determined in both hemispheres and compared to the control population. No significant differences in metabolite ratios were detected between epileptic and control dogs. However, higher values of NAA/Cho were noted in closer proximity to the last seizure whereas the Cho/NAA values were lower in dogs with recent seizures, suggesting that these changes may be temporary (17).

A recent prospective, case–control study assessed and compared MRS spectra within the thalamus of healthy dogs and dogs with IE, focusing on NAA and Glx as well as comparing differences in the spectral data of IE dogs with and without ASMs (39). This study found reduced NAA/Cr in IE dogs on ASMs compared to both healthy controls and IE dogs not on ASMs, as well as reduced Glx/Cr values in IE dogs on ASMs therapy compared to dogs without. In humans with generalized tonic–clonic epileptic seizures, reductions in NAA were more severe in those experiencing more seizures and therefore, in this population of dogs with IE, this finding may reflect what is observed in people (39, 48, 49).

### Magnetic resonance elastography

Magnetic resonance elastography (MRE) is a rapidly developing imaging technique used to quantitatively assess the mechanical properties of tissues *in vivo.* As such, it is considered an image-based counterpart to palpation, commonly used by both physicians and veterinarians to diagnose and characterize diseases. MRE obtains information about tissue stiffness by using a special MRI technique involving three steps: (1) generating shear waves in the tissue, (2) acquiring phase-sensitive MR images to detect the propagating waves, and (3) processing the images to generate quantitative stiffness maps, called elastograms (20, 50) (Figures 2C–E).

Only 1 study has investigated brain viscoelastic changes in people with epilepsy. Huesmann et al. performed MRE in people with mesial temporal lobe epilepsy (MTLE) compared to healthy volunteers and found the hippocampus ipsilateral to the epileptogenic regions was stiffer than the contralateral part, and the stiffness ratio between hemispheres was higher in patients with MTLE compared to healthy participants. This shows that MRE is not only applicable in epilepsy, but it may be sensitive to microstructural pathology which could be present before grossly observable changes in hippocampal volume, an established imaging biomarker in MTLE (51). To date, there is only a single published abstract on the capability of performing MRE in the canine cadaveric brain, which demonstrated variation in tissue stiffness across regions, with a mean whole brain tissue viscoelasticity ± standard error of 2.99 ± 0.30 kPA. Thus, showing promise for the use of this technique in veterinary neuroimaging (52).

### Perfusion weighted imaging

Perfusion-weighted imagined (PWI) allows the assessment of blood volume flow in the brain through the determination of cerebral blood volume (CBV), cerebral blood flow (CBF), mean transient time (MTT), time of arrival (T0) to the region of interest (ROI), and time to peak concentration (TPP) (8, 53, 54), though the use of an exogenous contrast medium tracer injected intravenously, such as a gadolinium chelate (53, 55).

In people with TLE, PWI findings demonstrate hyperperfusion during ictus and hypoperfusion postically (31, 56–61). There are two canine IE studies, in which PWI was assessed interictally with similar findings to what has been documented in people. Hartmann et al. demonstrated that dogs with IE have decreased brain perfusion compared to healthy dogs (54). Nagendran et al. noted various changes in perfusion with some IE dogs demonstrating hyperperfusion and others having hypoperfusion.

### Arterial spin labeling

Arterial spin labeling (ASL) allows the quantification of brain perfusion (CBF) non-invasively, without the use of any contrast agent or ionizing radiation. Blood water entering the brain is magnetically labeled as an exogenous tracer (62, 63). The perfusion signal is obtained by subtracting the labeled image from a control image in which blood has not been labeled, resulting in an image with the signal intensity proportional to CBF (i.e., reflecting the amount of blood delivered to each voxel). This technique has been applied in people in the assessment of TLE and frontal lobe epilepsy, and in presurgical planning in MRI-negative focal epileptic patients (62–66).

In animals, brain ASL-MRI has been used widely in experimental research (67–71). Recently, Hoffman et al. successfully performed ASL in a large population of dogs and cats with a success rate of 95% in animals with a normal brain MRI. Future directions would include the application of this technique to potentially characterize various brain diseases in dogs and cats, including epilepsy (72).

### Voxel based morphemtry

Voxel-based morphometry (VBM) is an automatically computational quantitative MRI analysis technique used to detect differences in brain morphology across different subjects (73, 74). In people with TLE, VBM has demonstrated a reduced or increased volume of several gray matter structures, typically ipsilateral to the side of seizure onset (73).

There are limited volumetric studies in veterinary medicine, largely based on the significant variation in head and brain shape between breeds (75). Recently, Frank et al. assessed differences in gray matter volume (GVM) between healthy and epileptic Beagles and found a significant reduction in GMV in several areas of the brain in epileptic dogs (74).

### Functional MRI

Functional MRI (fMRI) is the representative functional imaging technique in human neuroscience and utilizes magnetic susceptibilities of oxyhemoglobin and deoxyhemoglobin (blood oxygen level-dependent [BOLD] contrast). Areas with increased brain activity have a greater metabolic demand and thus more oxyhemoglobin. fMRI has been performed in humans with TLE and often combined with electroencephalography (EEG) (8, 76, 77). Resting-state fMRI (rs-fMRI) is based on low-frequency fluctuations in the BOLD signal when the brain is at rest (78). In human epilepsy, rs-fMRI has demonstrated altered functional connectivity in large-scale networks, including attentional (79), perceptual (80), and default mode (81–83); with the most widely studied network in epilepsy being the default mode network (DMN) (84).

To date, the only awake fMRI studies in canine patients have been experimental and require extensive training for the patients to remain immobile, leading to the use of a small number of dogs (85–87). There is limited research on the use of rs-fMRI in diagnosing canine IE, but one study found significantly increased functional connectivity in the anterior DMN in dogs with IE compared to healthy controls (88).

## Limitations

Many of these modalities have yet to be established in the canine brain, and more work is needed to optimize these sequences for use in veterinary patients. Many of these techniques require technical skills, which significantly limits their integration into clinical practice in both veterinary and human medicine. Automated software for brain extraction and data processing has been developed in human medicine. A limitation in veterinary medicine is the highly variable anatomy of the canine brain (5, 89). Milne et al. assessed three different atlas-based segmentation techniques in the three basic canine brain shapes (brachycephalic, mesaticephalic, and dolichocephalic) (75). This study demonstrated that the use of manual brain extraction techniques with the application of brain shape-specific templates is highly accurate and repeatable. A limitation of this study is that while the MRIs were structurally normal, some of the patients enrolled had neurologic signs or behavioral changes. Therefore the effect of any undetected structural pathology cannot be determined (75). Further work is needed in developing automated brain extraction techniques in the canine brain.

## Conclusion

Canine IE is often a diagnosis of exclusion, and in most patients, potential changes associated with epileptic seizure activity may be missed on routine MRI. Therefore, more novel imaging techniques may be required to detect lesions in patients with structurally normal brains visualized with traditional MRI. These novel techniques provide clinicians and researchers opportunities to improve diagnostic capabilities and expand knowledge of targeted therapeutic planning and monitoring in both human and canine epilepsy.

## Author contributions

KF: Supervision, Writing – original draft, Writing – review & editing. AB: Writing – original draft, Writing – review & editing.
